# 3D modeling and fitted sphere analysis-based biotechniques unveil novel indicators for subacromial impingement syndrome

**DOI:** 10.3389/fbioe.2025.1709322

**Published:** 2026-01-05

**Authors:** Dongqiang Yang, Yanlong Liu, Yanbo Wang, Zhenyu Zhang, Biao Guo, Songsong Wei, Yong Hu

**Affiliations:** 1 Department of Orthopedics, The First Affiliated Hospital of Anhui Medical University, Hefei, China; 2 Department of Orthopaedics,Sports Medicine and Arthroscopy, Fuyang People’s Hospital Affiliated to Anhui Medical University, Anhui, China; 3 Department of Orthopedics, The Second People’s Hospital of Fuyang, Fuyang, China

**Keywords:** shoulder impingement syndrome, 3D modeling, fitted sphere, pseudo-moving domain, novel indicators

## Abstract

**Objective:**

To investigate the impact and diagnostic value of novel indicators based on 3D modeling and fitted sphere analysis for Subacromial Impingement Syndrome (SIS).

**Methods:**

CT data from patients with Subacromial Impingement Syndrome and healthy individuals were imported into a software system to reconstruct a 3D model of the index shoulder. The following parameters were measured: humeral head-pseudo-moving domain volume index, pseudo-moving domain anteversion angle, pseudo-moving domain abduction angle, Critical Shoulder Angle (CSA), Acromion Index (AI), Acromio-humeral Interval (AHI), Lateral Acromial Angle (LAA), Acromion-Greater Tuberosity Impingement Index (ATI), glenoid inclination angle, and glenoid ante/retroversion angle. Influencing factor analysis, Receiver Operating Characteristic curve analysis, and correlation analysis were then performed.

**Results:**

The humeral head-pseudo-moving domain volume index, pseudo-moving domain anteversion angle, and pseudo-moving domain abduction angle were all indicators for SIS (P < 0.05). The humeral head-pseudo-moving domain volume index had high accuracy in predicting SIS (AUC = 0.778, P < 0.001), with an optimal threshold of 0.690, at which sensitivity and specificity were 67.0% and 77.0%, respectively. The pseudo-moving domain anteversion angle had no diagnostic value. The pseudo-moving domain abduction angle had high accuracy in predicting SIS (AUC = 0.728, P < 0.001), with an optimal threshold of 75.012, at which sensitivity and specificity were 62.2% and 72.8%, respectively. When the new indicators were used in combination, the accuracy was higher (AUC = 0.859, P < 0.001), with optimal thresholds of 0.528 or 0.542. The humeral head-pseudo-moving domain volume index had correlation with AI, AHI, LAA, and ATI. The pseudo-moving domain anteversion angle showed no correlation with glenoid anteversion/retroversion. The pseudo-moving domain abduction angle had correlation with critical shoulder angle and glenoid superior inclination.

**Conclusion:**

The new indicators based on 3D modeling and fitted sphere analysis are indicators for SIS. The humeral head-pseudo-moving domain volume index and pseudo-moving domain abduction angle have prognostic value for SIS.

## Introduction

Subacromial Impingement Syndrome (SIS) is a common disorder characterized by a series of symptoms such as shoulder pain and dysfunction (Dong et al., 2024; Chaimongkhol et al., 2020), accounting for 44%–65% of patients with shoulder pain in clinical practice (Abu El Kasem et al., 2024). Relevant studies have shown that SIS can cause rotator cuff tears (Borbas et al., 2022), thereby severely affecting patients’ shoulder joint function and quality of life (Neer, 1972; Ozaki et al., 1988; Keshvari et al., 2024; Pasin and Pasin, 2021). Therefore, early diagnosis and personalized prevention and treatment of SIS are particularly important.

Currently, the diagnostic criteria for SIS still have certain limitations, mainly relying on clinical manifestations, physical examinations, and imaging assessments (Hanchard et al., 2013; Lorusso et al., 2021; Buss et al., 2009; Guosheng et al., 2017; Chen et al., 2016). Specifically, imaging assessments mostly rely on 2D planar measurement parameters (Hamid et al., 2012), such as the critical shoulder angle (CSA) (Moor et al., 2013), acromion index (AI) (Nyffeler et al., 2006), Acromio-humeral interval (AHI) (Neer, 1972), and acromion-greater tuberosity impingement index (ATI) (Liu et al., 2020). These measured values are significantly affected by factors such as patient positioning and X-ray techniques, and have inherent limitations in describing the dynamic 3D spatial relationship between the acromion and the humeral head (Kocadal et al., 2022). Therefore, finding more sensitive and specific assessment indicators is of great significance for improving the diagnostic accuracy and personalized prevention and treatment of SIS.

In recent years, breakthroughs in computer-aided 3D modeling technology have provided new avenues for studying the bony morphology of SIS (Chen et al., 2024; Ma et al., 2021; Chen et al., 2022; Li et al., 2025). Based on this technique, we simulated a region of humeral head movement, which we named the pseudo-moving domain (PMD). We innovatively proposed new evaluation metrics for SIS: the humeral head-pseudo-moving domain volume index, pseudo-moving domain anteversion angle, and pseudo-moving domain abduction angle. This study aims to use shoulder joint CT scan data from healthy and SIS populations to investigate whether these novel reference metrics are prognostic factors for SIS, as well as their diagnostic value.

## Materials and methods

### General information

Inclusion criteria for SIS patients: Meeting three of the following five criteria: (1) Tenderness at the anterolateral edge of the acromion; (2) Positive painful arc sign during upper limb abduction; (3) Significant pain during active shoulder movement compared to passive movement; (4) Positive Neer impingement test; (5) Presence of acromial spurs, partial or full-thickness rotator cuff tears (Wülker et al., 2001).

Exclusion criteria for SIS patients: (1) History of previous shoulder surgery or trauma; (2) Concomitant fractures or dislocations around the shoulder joint; (3) Incomplete or unavailable preoperative shoulder joint CT data.

Inclusion criteria for healthy individuals: (1) Undergoing a physical examination at our physical examination center, including a chest CT scan; (2) Having a chest CT with data that can be used to reconstruct a complete 3D shoulder model.

Exclusion criteria for healthy individuals: (1) Tenderness at the anterolateral edge of the acromion; (2) Positive painful arc sign during upper limb abduction; (3) Significant pain during active shoulder movement compared to passive movement; (4) Positive Neer impingement test; (5) Acromial spurs, partial or full-thickness rotator cuff tears; (6) History of previous shoulder surgery or trauma; (7) Concomitant fractures or dislocations around the shoulder joint. For healthy participants, the side of the shoulder joint chosen for analysis depended on the completeness of the shoulder CT data. If both sides met the inclusion criteria, one side was randomly selected for analysis (Li et al., 2025).

This study included a total of 100 patients with shoulder impingement syndrome who were treated at our hospital from January 2022 to June 2024, and 100 healthy individuals randomly selected by simple random sampling. All patients signed an informed consent form, and the study was approved by our institutional review board. See Table 1 for general subject data.

**TABLE 1:** General characteristics of subjects M (Q1, Q3).

	Healthy (n = 100)	SIS (n = 100)	Z value/χ^2^ value	P value
Age (years)	56.50 (51.25, 65.00)	56.00 (51.00, 64.00)	−0.466	0.641
Gender (male/female)	49/51	37/63	2.938	0.087
Side (left/right)	42/58	33/67	1.728	0.189

### Indicators

The imaging observation indicators for SIS in this study included: humeral head-pseudo-moving domain volume index, pseudo-moving domain anteversion angle, pseudo-moving domain abduction angle, CSA, AI, AHI, lateral acromial angle (LAA), ATI, glenoid superior inclination, and glenoid anteversion/retroversion angle (anteversion expressed as a positive value, while retroversion as a negative).

### Step-by-step measurements

**FIGURE 1:**
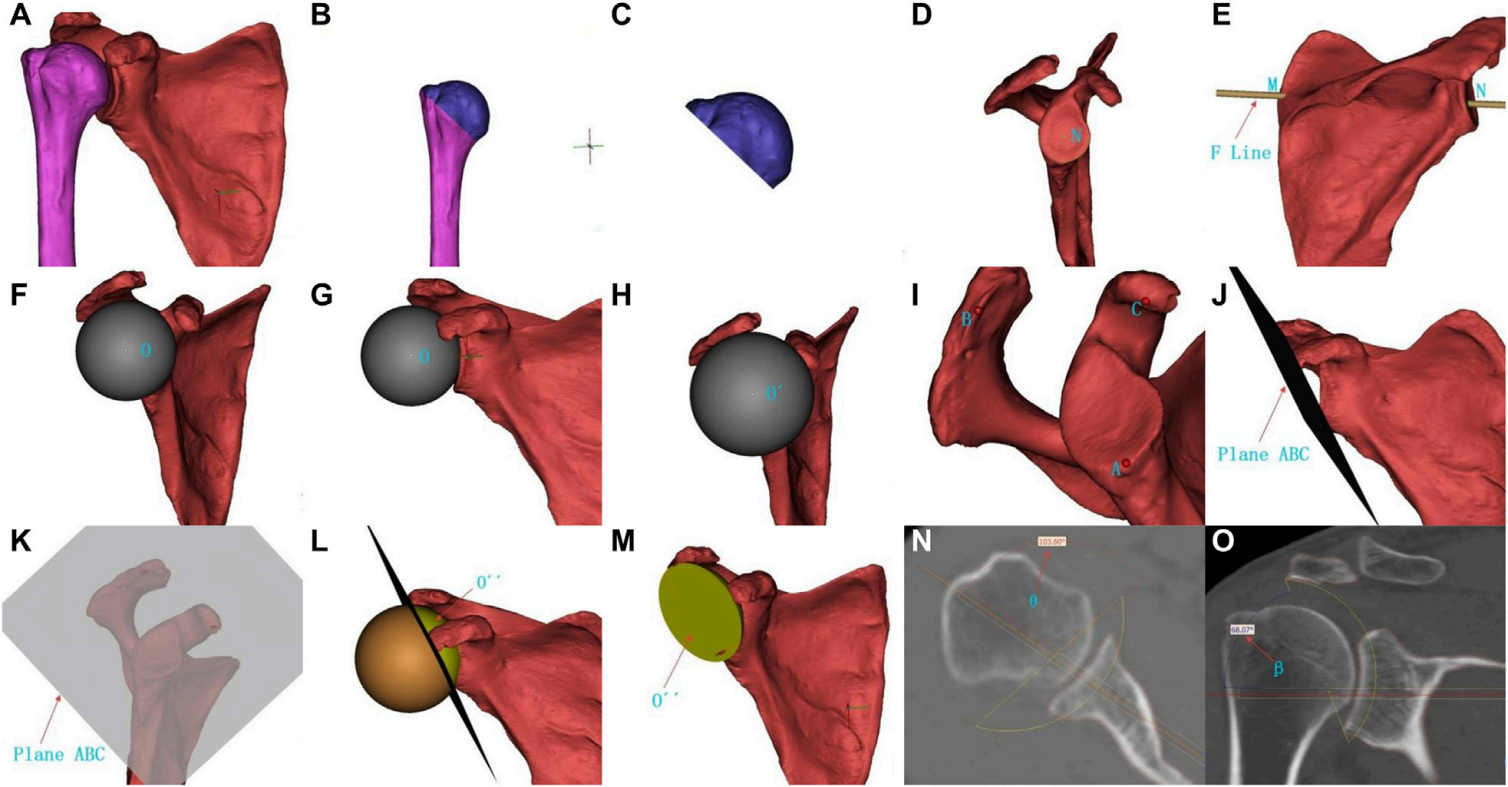
Measurement method for humeral head-pseudo-moving domain volume index, pseudo-moving domain anteversion angle, and pseudo-moving domain abduction angle. **(A)** Reconstructed 3D model of the shoulder joint; **(B–C)** Cutting and separating the humeral head; **(D)** Center of the glenoid N; **(E)** Most medial point of the scapula M, center of the glenoid N, line connecting the two points F line MN; **(F–G)** Original fitted sphere O; **(H)** Enlarged new fitted sphere O´; **(I)** Lowest point of the glenoid A, outermost point of the acromion B, medial apex of the coracoid process C; **(J–K)** Plane ABC established by the three points; **(L-M)** Spherical cap part O´´ near the glenoid side after cutting (pseudo-moving domain); **(N)** Angle θ; **(O)** Pseudo-moving domain abduction angle β.

**FIGURE 2:**
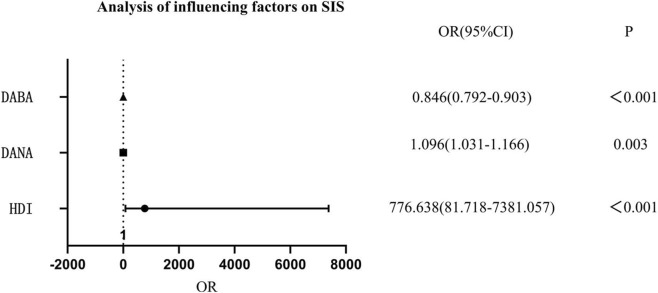
Analysis of influencing factors of HDI, DANA, DABA on SIS. Note: HDI, humeral head-pseudo-moving domain volume index; DANA, pseudo-moving domain anteversion angle; DABA, pseudo-moving domain abduction angle.

The target joint data of the German Siemens 64-row CT scan (tube voltage 120 kV, tube electrical current 300 mA, scanning layer thickness 0.6 mm) were used for 3D virtual reality and multi-planar reconstruction. CT data in DICOM format were imported into 3D simulation software (9yuan3D Digital Orthopedics System, Shanghai Jiao Tong University 3D Printing Innovation Research Center) to reconstruct a 3D model of the shoulder joint by using the threshold segmentation method (Figure 1A). The measurement steps were as follows: ① The humerus was cut from the apex of the greater tuberosity to the lower edge of the anatomical neck to separate the humeral head, and the volume of the humeral head V1 was recorded (Figures 1B,C); ② The center of the glenoid N was identified (Figure 1D), and a line MN was drawn from the most medial point of the scapula M to the center of the glenoid N (Figure 1E), which is the F line (Friedman line) (Kwon et al., 2005); ③ The humerus was hidden, and a fitted sphere O was created with the maximum radius of curvature of the glenoid (Figures 1F,G), then it was gradually enlarged by changing the diameter of the sphere until it contacted the coracoid process and acromion, obtaining a new fitted sphere O´ (Figure 1H); ④ A plane ABC was created using the lowest point of the glenoid A, the outermost point of the acromion B, and the medial apex of the coracoid process C (Figures 1I–K), then the fitted sphere O′ was cut according to the plane ABC (Figure 1L), leaving the spherical cap part O´´ near the glenoid side (Figure 1M), which is the pseudo-moving domain—a simulated area of humeral head activity, and its volume V2 was recorded; ⑤ The angle θ was measured according to the F line, and the pseudo-moving domain anteversion angle α (θ-90°) was calculated (Figure 1N); the abduction angle β was measured according to the F line (Figure 1O); ⑥ Calculate the humeral head-pseudo-moving domain volume index: V1/V2.

### Statistical analysis

SPSS 26.0 statistical software was used to analyze the data. Binary Logistic regression analysis was used for influencing factor analysis of the new indicators, and GraphPad Prism 10 was used to draw forest plots. Receiver Operating Characteristic (ROC) curve analysis was performed for the new indicators and the regression prediction model. The Kolmogorov-Smirnov method was used for normality testing of the data. Pearson correlation analysis was used for normally distributed continuous variable data, which were expressed as mean ± standard deviation (SD). Spearman rank correlation analysis was used for non-normally distributed continuous variable data, and measurement data were expressed as median and interquartile range M (Q1, Q3). Chi-square test was used for comparison between groups of count data. P < 0.05 was considered statistically significant.

## Results

### Influencing factors related to SIS

The humeral head-pseudo-moving domain volume index, pseudo-moving domain anteversion angle, and pseudo-moving domain abduction angle were all influencing factors for SIS (P < 0.001, P = 0.003, P < 0.001, respectively). The humeral head-pseudo-moving domain volume index and pseudo-moving domain anteversion angle were risk factors for SIS, while the pseudo-moving domain abduction angle was a protective factor for SIS. For every 1-unit increase in the humeral head-pseudo-moving domain volume index, the risk of SIS increased significantly. For every 1° increase in the pseudo-moving domain anteversion angle, the risk of SIS increased by 9.6%. For every 1° increase in the pseudo-moving domain abduction angle, the risk of SIS decreased by 15.4%. See Figure 2.

### Measurements for predicting SIS

**FIGURE 3:**
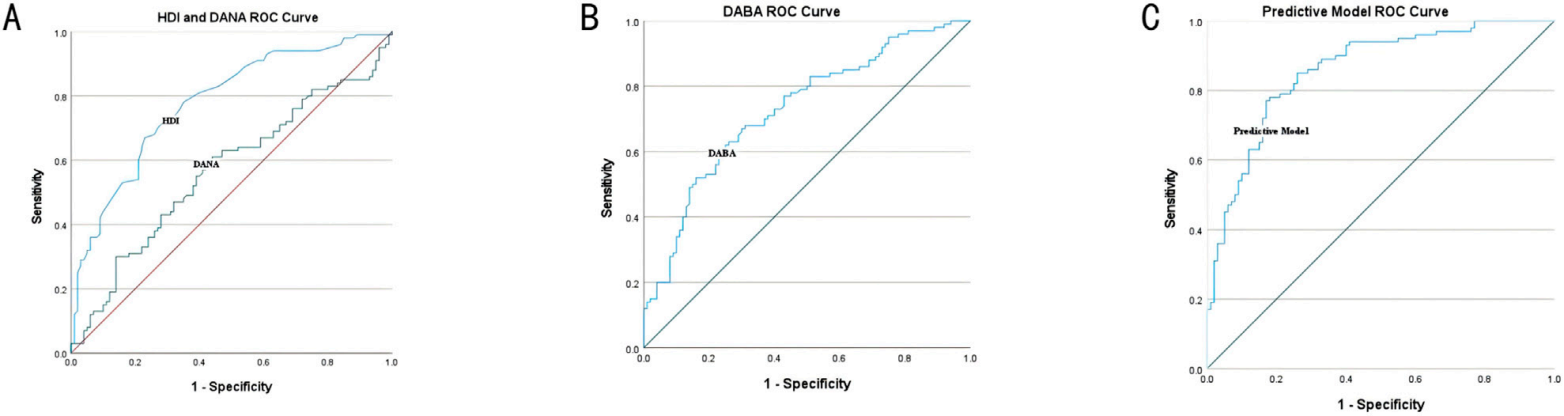
Receiver Operating Characteristic (ROC) curve analysis of HDI and DANA **(A)**: HDI, humeral head-pseudo-moving domain volume index; DANA, pseudo-moving domain anteversion angle. Receiver Operating Characteristic (ROC) curve analysis of DABA **(B)**: DABA, pseudo-moving domain abduction angle. Receiver Operating Characteristic (ROC) curve analysis of Predictive Model **(C)**.

The humeral head-pseudo-moving domain volume index had high accuracy in predicting SIS (AUC = 0.778, P < 0.001), with an optimal threshold of 0.690, at which sensitivity and specificity were 67.0% and 77.0%, respectively. The pseudo-moving domain anteversion angle had no diagnostic value for predicting SIS (AUC = 0.567, P = 0.104, P > 0.05), with an optimal threshold of 12.370, at which sensitivity and specificity were 61.0% and 56.0%, respectively. The pseudo-moving domain abduction angle had high accuracy in predicting SIS (AUC = 0.728, P < 0.001), with an optimal threshold of 75.012, at which sensitivity and specificity were 62.2% and 72.8%, respectively. When the new indicators were used in a combined predictive model, the accuracy was higher (AUC = 0.859, P < 0.001), with optimal thresholds of 0.528 or 0.542, at which sensitivity and specificity were 78.0% and 82.0% or 77.0% and 82.0%, respectively. See Figure 3; Table 2.

**TABLE 2:** Receiver operating characteristic (ROC) curve analysis.

Parameter	Cutoff value	AUC	P value	Sensitivity	Specificity
AHD	>0.690	0.778 (0.714–0.842)	<0.001	67.0%	77.0%
DANA	>12.370	0.567 (0.486–0.647)	0.104	61.0%	56.0%
BABA	>75.012	0.728 (0.659–0.798)	<0.001	62.2%	72.8%
Predictive model	>0.528	0.859 (0.807–0.910)	<0.001	78.0%	82.0%
Predictive model	>0.542	0.859 (0.807–0.910)	<0.001	77.0%	82.0%

HDI, humeral head-pseudo-moving domain volume index; DANA, pseudo-moving domain anteversion angle; DABA, and pseudo-moving domain abduction angle.

### Measurement correlation to SIS

**FIGURE 4:**
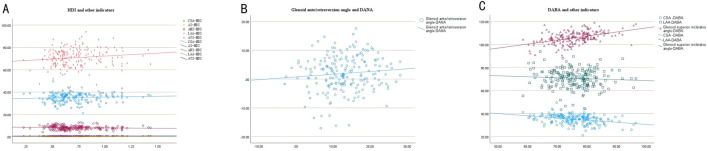
Correlation scatter plot of HDI and CSA, AI, AHI, LAA, ATI **(A)**: HDI, humeral head-pseudo-moving domain volume index; CSA, critical shoulder angle; AI, acromion index; AHI, acromio-humeral interval; LAA, lateral acromial angle; ATI, acromion-greater tuberosity impingement index. Correlation scatter plot of Glenoid ante/retroversion angle and DANA **(B)**: DANA, pseudo-moving domain anteversion angle. Correlation scatter plot of DABA and CSA, LAA, Glenoid superior inclination angle **(C)**: DABA,pseudo-moving domain abduction angle; CSA, critical shoulder angle; LAA, lateral acromial angle.

The humeral head-pseudo-moving domain volume index showed a certain correlation with AI, AHI, LAA, and ATI, with correlation coefficients of 0.181 (P = 0.010, P < 0.05), −0.143 (P = 0.044, P < 0.05), 0.151 (P = 0.032, P < 0.05), and 0.203 (P = 0.004, P < 0.05), respectively; however, it showed no correlation with CSA, with a correlation coefficient of 0.088 (P = 0.216, P > 0.05). The pseudo-moving domain anteversion angle showed no correlation with glenoid anteversion/retroversion, with a correlation coefficient of 0.098 (P = 0.167, P > 0.05). The pseudo-moving domain abduction angle showed a certain correlation with CSA and glenoid superior inclination, with correlation coefficients of −0.316 (P < 0.001) and 0.471 (P < 0.001), respectively; however, it showed no correlation with the abduction angle (LAA), with a correlation coefficient of −0.072 (P = 0.313, P > 0.05). See Figure 4; Table 3.

**TABLE 3:** Correlation analysis of humeral head-pseudo-moving domain volume index, pseudo-moving domain anteversion angle, pseudo-moving domain abduction angle, and other indicators.

	Correlation coefficient	P value
CSA and HDI	0.088	0.216
AI and HDI	0.181	0.010
AHI and HDI	−0.143	0.044
LAA and HDI	0.151	0.032
ATI and HDI	0.203	0.004
Glenoid ante/retroversion and DANA	0.098	0.167
CSA and DABA	−0.316	<0.001
LAA and DABA	−0.072	0.313
Glenoid superior inclination and DABA	0.471	<0.001

HDI, humeral head-pseudo-moving domain volume index; DANA, pseudo-moving domain anteversion angle; DABA, and pseudo-moving domain abduction angle; CSA, critical shoulder angle; AI, acromion index; AHI, Acromio-humeral interval; LAA, lateral acromial angle; ATI, acromion-greater tuberosity impingement index.

## Discussion

This study proposes three new metrics for the diagnosis of SIS. The results indicate that the humeral head-pseudo-moving domain volume index and the pseudo-moving domain anteversion angle are risk factors for SIS, while the pseudo-moving domain abduction angle is a protective factor. Both the humeral head-pseudo-moving domain volume index and the pseudo-moving domain abduction angle can effectively diagnose SIS with high sensitivity and specificity.

SIS is common in middle-aged and elderly individuals, but its prevalence is increasing in younger populations (Page, 2011). The pathogenesis remains not fully understood (Dorrestijn et al., 2009). The subacromial impingement theory suggests that a reduction in the subacromial space causes repeated impingement of subacromial tissues (Nizam Siron et al., 2021), leading to tissue damage, shoulder pain, and functional disability (Neer, 1972; Kwon et al., 2005). Other scholars believe that acromial morphology is significantly related to SIS (Barbier et al., 2013; Balke et al., 2013). At present, there are many methods for measuring acromial anatomy and morphology (Neer, 1972; Nyffeler et al., 2006), such as acromial classification, CSA, AI, AHI, LAA, and ATI. However, these methods all have limitations in diagnosing SIS. Our findings may provide a partial supplement to the diagnosis of SIS.

Shoulder can be divided into a broad joint and a narrow joint. The broad joint includes the glenohumeral, acromioclavicular, scapulothoracic, and subacromial joints, which are formed by soft tissues like muscles and ligaments (Yu et al., 2013; Petersilge et al., 1993); while the narrow shoulder joint is limited to the glenohumeral joint (Li et al., 2017; Moor et al., 2014). We found that the shoulder and hip joints are structurally similar, both being ball-and-socket joints. The key difference is that the hip’s acetabulum is a deep socket with more bony structures, while the shoulder’s glenoid is shallower, allowing for a greater range of motion (Garving et al., 2017).

The impingement theory, as proposed by Neer (1972), suggests that impingement primarily occurs at the anterior one-third of the inferior acromial surface and the coracoacromial ligament. However, SIS is not limited to the acromion, and impingement can occur in various directions (Garving et al., 2017). We hypothesized that the shoulder joint might have an unseen “socket” that matches the humeral head to accommodate its movement. When there is a mismatch between the humeral head and this “socket,” impingement may occur. This study simulated a spherical socket for the shoulder joint using 3D modeling. A fitted sphere was created based on the maximum curvature radius of the glenoid and then expanded until it made contact with the coracoid process and the acromion (Ghafurian et al., 2016). By using the plane defined by the lowest point of the glenoid, the most lateral point of the acromion, and the medial vertex of the coracoid process, we sectioned the fitted sphere to create the glenoid-side spherical cap. This portion, which we named the pseudo-moving domain, simulates the active area of the humeral head. We noticed the pseudo-moving domain has a morphology similar to the acetabulum, and abnormal acetabular anteversion and abduction angles are known risk factors for femoroacetabular impingement syndrome (Cengiz et al., 2021; Bai et al., 2021; Murphy et al., 2021). Therefore, we proposed that the morphology of the pseudo-moving domain, specifically its anteversion and abduction angles, may influence the degree of containment of the humeral head and could be a factor related to SIS. We used the Friedman line, which connects the most medial point of the scapula to the center of the glenoid, to measure the pseudo-moving domain inclination angles (Kwon et al., 2005).

To link the metrics to the humeral head, we created the humeral head-pseudo-moving domain volume index, a ratio of the humeral head volume to the pseudo-moving domain volume, referencing AHI and ATI. Through data analysis of healthy individuals and SIS patients, we found that the humeral head-pseudo-moving domain volume index, pseudo-moving domain anteversion angle, and pseudo-moving domain abduction angle are all influencing factors for SIS.

When these three indicators are used in combination, the accuracy of the SIS predictive model can reach 85.9%, with high sensitivity and specificity.

This study found that although we used the glenoid curvature radius to fit a sphere (Ghafurian et al., 2016), there are some differences in the scapula itself. During the process of enlarging the fitted sphere, its contact with the acromion and coracoid process showed two modes: sequential or simultaneous contact. In samples with sequential contact, when one position was contacted, the other was also nearly contacted, so it could be considered approximately simultaneous contact, which did not cause significant interference to the measurement results. According to the author’s analysis, the differences in the sphere-fitting process may provide some clues about the location of impingement, such as anterior coracoid impingement or posterior acromial edge impingement (Barbu et al., 2020; Ertekin and Kasar, 2021).

Based on the humeral head-pseudo-moving domain volume index, we found that its relatively suitable range is 0.41–0.78. When the index is greater than this range, it suggests that the humeral head has a small range of motion under the acromion, similar to femoroacetabular impingement caused by excessive acetabular anteversion or over-coverage, which increases the chance of impingement. When the index is less than this range, it suggests instability during humeral head movement, which can still lead to impingement. The pseudo-moving domain anteversion and abduction angles may also have a suitable range. An anteversion angle greater than the suitable range could lead to easier impingement of the bone at the posterior edge of the acromion, while an abduction angle smaller than the suitable range could increase the chance of bone impingement at the lateral acromial position, but this inference requires further study. CSA was first proposed by Moor et al. (2013), and is defined as the angle between the line from the inferolateral point of the acromion to the inferior margin of the glenoid and the line connecting the superior and inferior margins of the glenoid on a standard anteroposterior X-ray. Its safe range is generally considered to be between 30° and 35° (Moor et al., 2013; Scheibel et al., 2016). Studies have shown that a high CSA is an important risk factor for shoulder impingement syndrome and has a certain accuracy in predicting rotator cuff injury (Garofalo et al., 2011; Umer et al., 2012). However, as research on CSA has deepened, the controversy has grown, and using CSA alone is not a reliable method for diagnosing SIS. During our measurements, we found that in the included SIS population, 16 patients had a normal CSA, indicating that SIS can still occur even if the CSA is within the safe range. In the healthy population, 35 people also had a CSA greater than 35°. The researchers analyzed that the pseudo-moving domain, which simulates the range of motion of the humeral head, includes the acromial morphology involved in CSA, the length of the acromion extending laterally, the downward sloping angle, and the upward inclination angle of the glenoid. Notably, CSA is a two-dimensional measurement and is limited to the effect of acromial morphology in the coronal and transverse planes on the range of motion of the humeral head. It cannot reflect the effect of the acromion’s morphological features in the sagittal plane on the range of motion of the humeral head. However, the pseudo-moving domain includes the effect of acromial morphology on the range of motion of the humeral head in the anteroposterior view. Therefore, the pseudo-moving domain abduction angle in this study also showed a certain correlation with CSA and the glenoid inclination angle. Our results showed no correlation between the humeral head-pseudo-moving domain volume index and CSA, which further illustrates the limitations of CSA.

The lateral side of the acromion may slope downward (Vaz et al., 2020) in the sagittal or coronal plane. Banas et al. (1995). Measured the angle between the bone cortex below the acromion and the glenoid, naming it LAA, yet its use in SIS remains controversial. While it has a certain diagnostic value for SIS, it still only describes the influence in the coronal and sagittal planes and does not involve the transverse plane. The diagnosis of SIS should involve the entire range of motion and is not limited to the bone under the acromion, as impingement can occur in various directions, including subcoracoid and internal impingement (Chu et al., 2001; Hoenecke et al., 2008). Yao et al. (1996) and Tétreault et al. (2004) et al. also found that inter-observer consistency was poor, and the measurement of subacromial lateral tilting might be influenced by the observer. Like CSA, LAA only focuses on the scapula and ignores the crucial humeral head, so its value also has certain inherent limitations.

The AI proposed by Nyffeler et al. (2006) is the ratio of the vertical distance from the lateral edge of the acromion to the line connecting the superior and inferior margins of the glenoid and the vertical distance from the most lateral edge of the humeral head to the line connecting the superior and inferior margins of the glenoid. They believe that a larger AI is more likely to cause SIS, which can lead to degenerative wear of the shoulder joint and rotator cuff tears. However, AI is related to the motion state of the joint, and its value differs depending on the body position, so AI cannot completely evaluate SIS. The diagnostic value of AI values in different body positions needs further study. AHI is also known as “the second shoulder joint”. Its upper boundary is composed of the acromion, coracoid process, coracoacromial ligament, and acromioclavicular joint, and its lower boundary is the humeral head. The gap contains the supraspinatus tendon, infraspinatus tendon, long head of the biceps tendon, coracobrachialis ligament, and subacromial bursa (Neer, 1972; Chuang et al., 2021). The width of the subacromial space varies from person to person, with the average distance from the humeral head to the acromion being 9–10 mm. Related research shows that a smaller AHI increases the chance of SIS. Liu et al. (2020). Proposed the ATI, defined as the ratio of the distance from the center of rotation of the humeral head to the greater tuberosity and the distance from the center of rotation of the humeral head to the lower surface of the acromion. They believe that as ATI increases, the chance of SIS will increase. However, while the AI, AHI, and ATI are related to the humeral head, their measured values are not only significantly affected by factors such as patient position and X-ray but also fail to consider the effect of the anteroposterior diameter of the humeral head on shoulder joint movement. The humeral head-pseudo-moving domain volume index is originated from a 3D perspective. It may therefore be more advantageous in diagnosing SIS.

The results of the correlation analysis showed that there is no correlation between the pseudo-moving domain anteversion angle and the glenoid ante/retroversion angle. According to literature reports, the average inclination in the anteroposterior direction of the glenoid measured by 3D CT in normal people is −4.5°–2° (Lp and Agrawal, 2023; Goetti et al., 2020; Bakhsh and Nicandri, 2018). We believe this is because the glenoid ante/retroversion angle is relatively small and is masked when creating the fitted sphere, so the two do not show a significant correlation. Researchers believe that the size of the pseudo-moving domain anteversion angle has a certain suggestive effect on the location of impingement in SIS. For example, a larger pseudo-moving domain anteversion angle may suggest greater containment of the posterior bone of the acromion, causing the impingement location to be more biased towards the posterior part of the acromion, and the impingement location will extend to the posterior acromial bone. Compared with the traditional view that the impingement location is at the anterolateral corner of the acromion (Hardy et al., 2022), this may serve as a guide for the scope of acromioplasty.

Although the new indicators in this study rely on CT and carry a certain radiation risk, the CT data used for participants were not entirely derived from shoulder CT scans; some were from chest CT scans. Since the COVID-19 pandemic, the number of patients with pulmonary diseases has increased. Compared with conventional chest X-rays, CT offers advantages such as high resolution, full-lung coverage, three-dimensional quantification, and a higher detection rate of occult lesions. Moreover, advances in CT technology have significantly reduced radiation doses, and chest CT has gradually become a routine procedure in hospital admissions and physical examinations. This means that additional shoulder CT scans are not required, and the new indicators in this study can be measured using chest CT alone. At the same time, we have also noted research on MR technology in skeletal reconstruction (Su et al., 2025), but its accuracy is somewhat lower than that of CT modelling, and it is relatively costly. As technology advances and costs decrease, MR could also be applied to this study in the future.

This study has some limitations. First, despite two personnel being involved in the measurements, measurement errors could not be completely avoided. Second, this was a single-center study, and its generalizability needs to be verified through a larger sample, multi-center study. Additionally, the healthy individuals drawn from the physical examination center may not be entirely representative of the normal population and might include some asymptomatic individuals with SIS. Finally, the simulated humeral head range of motion is an approximation and does not perfectly represent the actual range of motion of the humeral head.

## Conclusion

The humeral head-pseudo-moving domain volume index, pseudo-moving domain anteversion angle, and pseudo-moving domain abduction angle, proposed based on 3D modeling and fitted sphere analysis, are all influencing factors for SIS. Both the humeral head-pseudo-moving domain volume index and the pseudo-moving domain abduction angle can effectively diagnose SIS, while the pseudo-moving domain anteversion angle has no diagnostic value. When these three metrics are used in combination, the diagnostic rate for SIS can be further improved, with high sensitivity and specificity.

## Data Availability

The raw data supporting the conclusions of this article will be made available by the authors, without undue reservation.

## References

[B1] Abu El Kasem S. T. Alaa F. A. A. Abd El-Raoof N. A. Abd-Elazeim A. S. (2024). Efficacy of mulligan thoracic sustained natural apophyseal glides on sub-acromial pain in patients with sub-acromial impingement syndrome: a single-blinded randomized controlled trial. J. Man. Manip. Ther. 32 (6), 584–593. 10.1080/10669817.2024.2341453 38618993 PMC11578423

[B2] Bai J. Hu X. Tan T. Hua T. Xia W. Jones C. (2021). The evaluation indicators of X-ray radiographs for femoroacetabular impingement syndrome. J. Med. Imaging Health Inf. 11, 557–562. 10.1166/jmihi.2021.3320

[B3] Bakhsh W. Nicandri G. (2018). Anatomy and physical examination of the shoulder. Sports Med. Arthrosc. Rev. 26 (3), e10–e22. 10.1097/JSA.0000000000000202 30059442

[B4] Balke M. Schmidt C. Dedy N. Banerjee M. Bouillon B. Liem D. (2013). Correlation of acromial morphology with impingement syndrome and rotator cuff tears. Acta Orthop. 84 (2), 178–183. 10.3109/17453674.2013.773413 23409811 PMC3639339

[B5] Banas M. P. Miller R. J. Totterman S. (1995). Relationship between the lateral acromion angle and rotator cuff disease. J. Shoulder Elb. Surg. 4 (6), 454–461. 10.1016/s1058-2746(05)80038-2 8665291

[B6] Barbier O. Block D. Dezaly C. Sirveaux F. Mole D. (2013). Os acromiale, a cause of shoulder pain, not to be overlooked. Orthop. Traumatol. Surg. Res. 99 (4), 465–472. 10.1016/j.otsr.2012.10.020 23644030

[B7] Barbu S. G. Nicoara A. D. Alistar D. E. Badea I. A. Mihai B. (2020). Subcoracoid impingement – a global view. 2. Walter de Gruyter GmbH. Ovidius University of Constanta. 10.2478/arsm-2020-0018

[B8] Borbas P. Hartmann R. Ehrmann C. Ernstbrunner L. Wieser K. Bouaicha S. (2022). Acromial morphology and its relation to the glenoid is associated with different partial rotator cuff tear patterns. J. Clin. Med. 12 (1), 233. 10.3390/jcm12010233 36615033 PMC9821296

[B9] Buss D. D. Freehill M. Q. Marra G. (2009). Typical and atypical shoulder impingement syndrome: diagnosis, treatment, and pitfalls. Instr. Course Lect. 58, 447–457. 19385554

[B10] Cengiz M. Keven A. Ztürk S. Salim H. Gölpınar M. Gökkuş K. (2021). The frequency of the bony parameters of femoroacetabular impingement syndrome in young asymptomatic individuals: a computed tomography study. Anat. Int. J. Exp. & Clin. Anat. 15 (2), 145–151. 10.2399/ana.21.990828

[B11] Chaimongkhol T. Benjachaya S. Mahakkanukrauh P. (2020). Acromial morphology and morphometry associated with subacromial impingement syndrome. Anat. Cell Biol. 53 (4), 435–443. 10.5115/acb.20.166 32963132 PMC7769113

[B12] Chen C. Pan Z. Zhang C. Liu C. Chen L. (2016). Clinical research on the efficiency of physical examinations used for diagnosis of subacromial impingement syndrome. Zhongguo Gu Shang 29 (5), 434–438. 27505960

[B13] Chen X. Liu C. Liang T. Ren J. Su S. Li P. (2022). *In vivo* anatomical research by 3D CT reconstruction determines minimum acromiohumeral, coracohumeral, and glenohumeral distances in the human shoulder: evaluation of age and sex association in a sample of the Chinese population. J. Pers. Med. 12 (11), 1804. 10.3390/jpm12111804 36579520 PMC9694460

[B14] Chen Y. Qin M. Pang L. Guo B. Zhang C. Tang X. (2024). Influence analysis of glenohumeral bone structure on anterior shoulder instability. Zhongguo Xiu Fu Chong Jian Wai Ke Za Zhi 38 (12), 1433–1438. 10.7507/1002-1892.202408035 39694831 PMC11655379

[B15] Churchill R. S. Brems J. J. Kotschi H. (2001). Glenoid size, inclination, and version: an anatomic study. J. Shoulder Elb. Surg. 10 (4), 327–332. 10.1067/mse.2001.115269 11517362

[B16] Chuang H. Hong C. Hsu K. Kuan F. C. Chiang C. H. Chen Y. (2021). Radiographic greater tuberosity spurs and narrow acromiohumeral intervals are associated with advanced retraction of the supraspinatus tendon in patients with symptomatic rotator cuff tears. JSES Int. 5 (1), 77–82. 10.1016/j.jseint.2020.09.015 33554169 PMC7846697

[B17] Dong W. Du K. Shi B. Wang T. Lu B. Hou Z. (2024). Distribution and analysis of subacromial spurs and the relationship with acromial classification and angle in healthy individuals. PLoS One 19 (3), e0301066. 10.1371/journal.pone.0301066 38547302 PMC10977877

[B18] Dorrestijn O. Stevens M. Winters J. C. van der Meer K. Diercks R. L. (2009). Conservative or surgical treatment for subacromial impingement syndrome? A systematic review. J. Shoulder Elb. Surg. 18 (4), 652–660. 10.1016/j.jse.2009.01.010 19286397

[B19] Ertekin E. Kasar Z. S. (2021). Does the coraco-acromial angle contribute to the diagnosis of impingement syndrome? Bagcilar Med. Bull. 6, 99–104. 10.4274/bmb.galenos.2020.11.079

[B20] Garofalo R. Conti M. Massazza G. Cesari E. Vinci E. Castagna A. (2011). Subcoracoid impingement syndrome: a painful shoulder condition related to different pathologic factors. Musculoskelet. Surg. 95 (1), 25–29. 10.1007/s12306-011-0142-7 21643947

[B21] Garving C. Jakob S. Bauer I. Nadjar R. Brunner U. H. (2017). Impingement syndrome of the shoulder. Dtsch. Arztebl Int. 114 (45), 765–776. 10.3238/arztebl.2017.0765 29202926 PMC5729225

[B22] Ghafurian S. Galdi B. Bastian S. Tan V. Li K. (2016). Computerized 3D morphological analysis of glenoid orientation. J. Orthop. Res. 34 (4), 692–698. 10.1002/jor.23053 26400654

[B23] Goetti P. Denard P. J. Collin P. Ibrahim M. Hoffmeyer P. Lädermann A. (2020). Shoulder biomechanics in normal and selected pathological conditions. EFORT Open Rev. 5 (8), 508–518. 10.1302/2058-5241.5.200006 32953136 PMC7484714

[B24] Guosheng Y. Chongxi R. Guoqing C. Junling X. Hailong J. (2017). The diagnostic value of a modified neer test in identifying subacromial impingement syndrome. Eur. J. Orthop. Surg. Traumatol. 27 (8), 1063–1067. 10.1007/s00590-017-1979-8 28534226

[B25] Hamid N. Omid R. Yamaguchi K. Steger-May K. Stobbs G. Keener J. D. (2012). Relationship of radiographic acromial characteristics and rotator cuff disease: a prospective investigation of clinical, radiographic, and sonographic findings. J. Shoulder Elb. Surg. 21 (10), 1289–1298. 10.1016/j.jse.2011.09.028 22217644 PMC3725773

[B26] Hanchard N. C. A. Lenza M. Handoll H. H. G. Takwoingi Y. (2013). Physical tests for shoulder impingements and local lesions of Bursa, tendon or labrum that may accompany impingement. Cochrane Database Syst. Rev. 2013 (4), CD007427. 10.1002/14651858.CD007427.pub2 23633343 PMC6464770

[B27] Hardy V. Rony L. Bachler J. Favard L. Hubert L. (2022). Does isolated arthroscopic anterior acromioplasty modify critical shoulder angle? Revue de Chir. Orthop. traumatologique 108, 103040. 10.1016/j.otsr.2021.103040 34389495

[B28] Hoenecke H. R. J. Hermida J. C. Dembitsky N. Patil S. D'Lima D. D. (2008). Optimizing glenoid component position using three-dimensional computed tomography reconstruction. J. Shoulder Elb. Surg. 17 (4), 637–641. 10.1016/j.jse.2007.11.021 18374607

[B29] Keshvari S. Bagheri S. Taherinia A. (2024). The effects of kinesiotape on the thickness of the long head of the biceps tendon in athletes with subacromial impingement syndrome. Sci. J. Rehabilitation Med. 13 (2), 306–321. 10.32598/sjrm.13.2.3041

[B30] Kocadal O. Tasdelen N. Yuksel K. Ozler T. (2022). Volumetric evaluation of the subacromial space in shoulder impingement syndrome. Orthop. Traumatol. Surg. Res. 108 (2), 103110. 10.1016/j.otsr.2021.103110 34649000

[B31] Kwon Y. W. Powell K. A. Yum J. K. Brems J. J. Iannotti J. P. (2005). Use of three-dimensional computed tomography for the analysis of the glenoid anatomy. J. Shoulder Elb. Surg. 14 (1), 85–90. 10.1016/j.jse.2004.04.011 15723018

[B32] Li X. Xu W. Hu N. Liang X. Huang W. Jiang D. (2017). Relationship between acromial morphological variation and subacromial impingement: a three-dimensional analysis. PLoS One 12 (4), e0176193. 10.1371/journal.pone.0176193 28441418 PMC5404845

[B33] Li M. Fan M. Zhang Y. Shao P. Liu T. Zuo J. (2025). A novel proportional method for the simplified assessment of glenoid bone loss in patients with anterior shoulder instability. Am. J. Sports Med. 53 (1), 24–32. 10.1177/03635465241294076 39741485

[B34] Liu H. Xu X. X. Xu D. L. Hu Y. Z. Pan X. Y. Yu Z. (2020). The acromion-greater tuberosity impingement index: a new radiographic measurement and its association with rotator cuff pathology. J. Orthop. Surg. Hong. Kong. 28 (1), 2309499020913348. 10.1177/2309499020913348 32212965

[B35] Lorusso M. Mastrangelo E. Garofalo G. Ristori D. Brindisino F. (2021). Diagnostic accuracy of physical tests and imaging techniques in patients with shoulder impingement syndrome. Muscles, Ligaments Tendons Journal 11, 383. 10.32098/mltj.03.2021.03

[B36] Lp M. R. L. Agrawal D. K. (2023). Biomechanical forces in the tissue engineering and regeneration of shoulder, hip, knee, and ankle joints. J. Biotechnol. Biomed. 6 (4), 491–500. 10.26502/jbb.2642-91280111 38037618 PMC10688570

[B37] Ma Q. Sun C. Du R. Liu P. Wu S. Zhang W. (2021). Morphological characteristics of acromion and acromioclavicular joint in patients with shoulder impingement syndrome and related recommendations: a three-dimensional analysis based on multiplanar reconstruction of computed tomography scans. Orthop. Surg. 13 (4), 1309–1318. 10.1111/os.13001 33955185 PMC8274212

[B38] Moor B. K. Bouaicha S. Rothenfluh D. A. Sukthankar A. Gerber C. (2013). Is there an association between the individual anatomy of the scapula and the development of rotator cuff tears or osteoarthritis of the glenohumeral joint? a radiological study of the critical shoulder angle. Bone Jt. J. 95-B (7), 935–941. 10.1302/0301-620X.95B7.31028 23814246

[B39] Moor B. K. Wieser K. Slankamenac K. Gerber C. Bouaicha S. (2014). Relationship of individual scapular anatomy and degenerative rotator cuff tears. J. Shoulder Elb. Surg. 23 (4), 536–541. 10.1016/j.jse.2013.11.008 24480324

[B40] Murphy N. Diamond L. Bennell K. (2021). Which hip morphology measures and patient factors are associated with age of onset and symptom severity in femoroacetabular impingement syndrome?. Hip International, 11207000211038550. 10.1177/11207000211038550 34424780

[B41] Neer C. S. N. (1972). Anterior acromioplasty for the chronic impingement syndrome in the shoulder: a preliminary report. J. Bone Jt. Surg. Am. 54 (1), 41–50. 10.2106/00004623-197254010-00003 5054450

[B42] Nizam Siron K. Mat Lani M. T. Low C. L. Kow R. Y. (2021). Arthroscopic subacromial decompression in the treatment of shoulder impingement syndrome: a prospective study in Malaysia. Cureus 13 (11), e19254. 10.7759/cureus.19254 34900455 PMC8648140

[B43] Nyffeler R. W. Werner C. M. L. Sukthankar A. Schmid M. R. Gerber C. (2006). Association of a large lateral extension of the acromion with rotator cuff tears. J. Bone Jt. Surg. Am. 88 (4), 800–805. 10.2106/JBJS.D.03042 16595470

[B44] Ozaki J. Fujimoto S. Nakagawa Y. Masuhara K. Tamai S. (1988). Tears of the rotator cuff of the shoulder associated with pathological changes in the acromion. A study in cadavera. J. Bone Jt. Surg. Am. 70 (8), 1224–1230. 10.2106/00004623-198870080-00015 3417708

[B45] Page P. (2011). Shoulder muscle imbalance and subacromial impingement syndrome in overhead athletes. Int. J. Sports Phys. Ther. 6 (1), 51–58. 21655457 PMC3105366

[B46] Pasin T. Pasin O. (2021). Assessment of quality of life in patients with subacromial impingement syndrome. J. Contemp. Med. (3). 10.16899/JCM.862632

[B47] Petersilge C. A. Witte D. H. Sewell B. O. Bosch E. Resnick D. (1993). Normal regional anatomy of the shoulder. Magn. Reson Imaging Clin. N. Am. 1 (1), 1–18. 10.1016/s1064-9689(21)00284-1 7584205

[B48] Scheibel M. Hug K. Gerhardt C. Krueger D. (2016). Arthroscopic reduction and fixation of large solitary and multifragmented anterior glenoid rim fractures. J. Shoulder Elb. Surg. 25 (5), 781–790. 10.1016/j.jse.2015.09.012 26652699

[B49] Su Z. Wang Y. Huang C. He Q. Lu J. Liu Z. (2025). Quantifying the trajectory of percutaneous endoscopic lumbar discectomy in 3D lumbar models based on automated MR image Segmentation-A cross-sectional study. Orthop. Surg. 17 (9), 2689–2698. 10.1111/os.70112 40745956 PMC12404874

[B50] Tétreault P. Krueger A. Zurakowski D. Gerber C. (2004). Glenoid version and rotator cuff tears. J. Orthop. Res. 22 (1), 202–207. 10.1016/S0736-0266(03)00116-5 14656681

[B51] Umer M. Qadir I. Azam M. (2012). Subacromial impingement syndrome. Orthop. Rev. (Pavia). 4 (2), e18. 10.4081/or.2012.e18 22802986 PMC3395987

[B52] Vaz A. Reifegerste C. P. Trippia C. R. Linhares L. S. Trindade F. B. Thomaz J. E. (2020). Effect of the acromial inferolateral tilt on subacromial impingement syndrome: a retrospective magnetic resonance imaging assessment. Radiol. Bras. 53 (6), 366–374. 10.1590/0100-3984.2019.0127 33304003 PMC7720662

[B53] Wülker N. Mansat M. Fu F. H. (2001). Shoulder surgery: an illustrated textbook. London: Imprint CRC Press. 10.3109/9780203417447

[B54] Yao L. Lee H. Y. Gentili A. Shapiro M. M. (1996). Lateral down-sloping of the acromion: a useful MR sign? Clin. Radiol. 51 (12), 0–872. 10.1016/s0009-9260(96)80085-7 8972653

[B55] Yu D. Turmezei T. D. Kerslake R. W. (2013). FIESTA: an MR arthrography celebration of shoulder joint anatomy, variants, and their mimics. Clin. Anat. 26 (2), 213–227. 10.1002/ca.22066 22431407

